# Supplementary material for: Convergent evolution of the ladder-like ventral nerve cord in Annelida
Source: Front Zool. 2018 Sep 27;15:36. doi: 10.1186/s12983-018-0280-y (PMC6161469; doi:10.1186/s12983-018-0280-y)
Supplement: Supplementary file 1 — Table S1. Sampling sites and fixation/preservation details. (DOCX 15 kb) [file 12983_2018_280_MOESM1_ESM.docx]

| **family** | **Species** | **Sampling site** | **Specimens preserved for…** | | | |
| --- | --- | --- | --- | --- | --- | --- |
|  |  |  | **RNA** | **Azan** | **TEM** | **CLSM** |
| **Ampharetidae** | ***Hypania invalida* (Grube, 1860)** | **river “Elbe” near Magdeburg, Germany** | **x** |  |  |  |
| **Amphinomidae** | ***Eurythoe complanata* (Pallas, 1766)** | **marine aquarium, Leipzig, Germany (Azan) and marine aquarium Osnabrück, Germany (TEM)** |  | **x** | **x** | **x** |
|  | ***Paramphimone* sp*.*** | **marine aquarium Osnabrück, Germany** |  |  | **x** |  |
| **Apistobranchidae** | ***Apistobranchus tullbergi* (Théel, 1879)** | **Quequertarsuaq, Disko Island, Greenland** | **x** | **x** | **x** | **x** |
| **Chaetopteridae** | ***Chaetopterus variopedatus* (Renier, 1804)** | **Morgat, France** |  | **x** |  |  |
|  | ***Spiochaetopterus costarum* (Claparède, 1869)** | **Poldouhan, France** |  | **x** | **x** |  |
|  | ***Phyllochaetopterus* sp*.*** | **marine aquarium, Leipzig, Germany** |  |  |  | **x** |
| **Cirratulidae** | ***Cirratulus cirratus* (O.F. Müller, 1776)** | **marine aquarium, Leipzig** | **x** |  |  |  |
| **Magelonidae** | ***Magelona mirabilis* (Johnston, 1865)** | **Morgat, France** |  |  | **x** | **x** |
|  | ***Magelona mirabilis* (Johnston, 1865)** | **Wimereux, France** |  | **x** |  |  |
|  | ***Magelona filiformis* Wilson, 1959** | **Morgat, France** |  |  |  | **x** |
| **Oweniidae** | ***Owenia fusiformis* Delle Chiaje, 1844** | **Saint-Efflam, France** |  |  | **x** | **x** |
|  | ***Owenia fusiformis* Delle Chiaje, 1844** | **Poldouhan, France** |  | **x** |  |  |
|  | ***Myriochele heeri* Malmgren, 1867** | **Quequertarsuaq, Disko Island, Greenland** | **x** |  |  | **x** |
|  | ***Myriowenia* sp.** | **New South Wales, Australia** |  | **x** |  |  |
| **Orbiniidae** | ***Scoloplos armiger* (Müller, 1776)** | **Roscoff, France** | **x** |  |  |  |
| **Psammodrilidae** | ***Psammodrilus aedificator* Kristensen & Nørrevang, 1982** | **Iterdla, Disko Island, Greenland** | **x** |  |  |  |
|  | ***Psammodrilus balanoglossoides* Swedmark, 1952** | **Ellekildehage, Oresund, Denmark** |  |  | **x** | **x** |
|  | ***Psammodrilus balanoglossoides* Swedmark, 1952** | **List, Sylt, Germany** | **x** | **x** |  |  |
|  | ***Psammodrilus curinigallettii* Worsaae, Martinez & Kvindeberg, 2015** | **Napoli, Italy** |  |  |  | **x** |
| **Sabellariidae** | ***Sabellaria alveolata* (Linnaeus, 1767)** | **Saint-Efflam, France** |  | **x** |  |  |
| **Scalibregmatidae** | ***Scalibregma inflatum* Rathke, 1843** | **Morlaix, France** | **x** |  |  |  |
| **Sipuncula** | ***Phascolosoma granulatum* Leuckart, 1828** | **Roscoff, France** | **x** |  |  |  |
| **Terebellomorpha** | ***Lanice conchilega* (Pallas, 1766)** | **Roscoff, France** | **x** |  |  |  |
| **Tomopteridae** | ***Tomopteris helgolandica* Greeff, 1879** | **Bergen, Norway** |  | **x** |  | **x** |
