# Supplementary material for: Convergent evolution of the ladder-like ventral nerve cord in Annelida
Source: Front Zool. 2018 Sep 27;15:36. doi: 10.1186/s12983-018-0280-y (PMC6161469; doi:10.1186/s12983-018-0280-y)
Supplement: Supplementary file 2 — Table S2. List of taxa used in the phylogenomic study and accession number. Species and accession numbers in bold were either newly sequenced or re-sequenced for deeper coverage in the present study. (DOCX 14 kb) [file 12983_2018_280_MOESM2_ESM.docx]

| **Higher Taxon** | **Species** | **Accession number** | **Data type** |
| --- | --- | --- | --- |
| **Mollusca** |  |  |  |
| Cephalopoda | *Lottia gigantea* | GCA_000327385.1 | Genome |
| Gastropoda | *Octopus bimaculoides* | GCA_001194135.1 | Genome |
| **Brachiopoda** |  |  |  |
| Linguliformea | *Lingula anatina* | GCA_001039355.1 | Genome |
| **Nemertea** |  |  |  |
| Palaeonemertea | *Cephalotrix linearis* | SRX534866 | Transcriptome |
| **Annelida** |  |  |  |
| Amphinomidae | *Eurythoe complanata* | tbd | Transcriptome |
|  | *Paramphinome jeffreysii* | SRX518630 | Transcriptome |
| Apistobranchidae | ***Apistobranchus tullbergi*** | SRX2848073 | Transcriptome |
| Arenicolidae | *Arenicola marina* | SRX1015734 | Transcriptome |
| Capitellidae | *Capitella teleta* | GCA_000328365.1 | Genome |
| Chaetopteridae | ***Chaetopterus variopedatus*** | SRX2848071 | Transcriptome |
|  | *Mesochaetopterus minutus* | SRX965459 | Transcriptome |
|  | *Phyllochaetopterus* sp. | SRX523008 | Transcriptome |
|  | *Spiochaetopterus typicus* | SRX513557 | Transcriptome |
| Cirratulidae | ***Cirratulus cirratus*** | SRX2848072 | Transcriptome |
| Clitellata | *Eisenia fetida* | SRX1483515 | Transcriptome |
|  | *Helobdella robusta* | GCA_000326865.1 | Genome |
| Echiura | *Bonellia viridis* | SRX1024222 | Transcriptome |
| Eunicidae | *Marphysa bellii* | SRX515220 | Transcriptome |
|  | *Eunice torquata* | SRX1015601 | Transcriptome |
| Magelonidae | *Magelona berkeleyi* | SRX522872 | Transcriptome |
|  | *Magelona johnstoni* | SRX512803 | Transcriptome |
|  | *Magelona pitelkai* | SRX1022769 | Transcriptome |
| Nephtyidae | *Nephtys caeca* | SRX515219 | Transcriptome |
| Nereididae | *Platynereis dumerilii* | dbEST* | Transcriptome |
| Orbiniidae | *Naineris dendritica* | SRX1024019 | Transcriptome |
|  | ***Scoloplos armiger*** | SRX2848069 | Transcriptome |
| Oweniidae | ***Myriochele heeri*** | SRX2848070 | Transcriptome |
|  | *Owenia fusiformis* | SRX512807 | Transcriptome |
|  | *Owenia* sp. | SRX1015948 | Transcriptome |
| Phyllodocidae | *Phyllodoce medipapillata* | SRX1023933 | Transcriptome |
| Psammodrilidae | ***Psammodrilus aedificator*** | SRX2848074 | Transcriptome |
|  | ***Psammodrilus balanoglossoides*** | SRX2848067 | Transcriptome |
| Sabellidae | *Megalomma vesicolosum* | SRX514826 | Transcriptome |
| Scalibgregmatidae | ***Scalibregma inflatum*** | SRX2848068 | Transcriptome |
| Siboglinidae | *Riftia pachyptila* | SRX098351 | Transcriptome |
| Sipuncula | *Phascolopsis gouldii* | SRX755857 | Transcriptome |
|  | ***Phascolosoma granulatum*** | SRX2848075 | Transcriptome |
| Spionidae | *Scolelepis squamata* | SRX512813 | Transcriptome |
| Terebellomorpha | ***Hypania invalida*** | SRX2848077 | Transcriptome |
|  | ***Lanice conchilega*** | SRX2848076 | Transcriptome |
