# Supplementary figures and images for: Convergent evolution of the ladder-like ventral nerve cord in Annelida
Source: Front Zool. 2018 Sep 27;15:36. doi: 10.1186/s12983-018-0280-y (PMC6161469; doi:10.1186/s12983-018-0280-y)

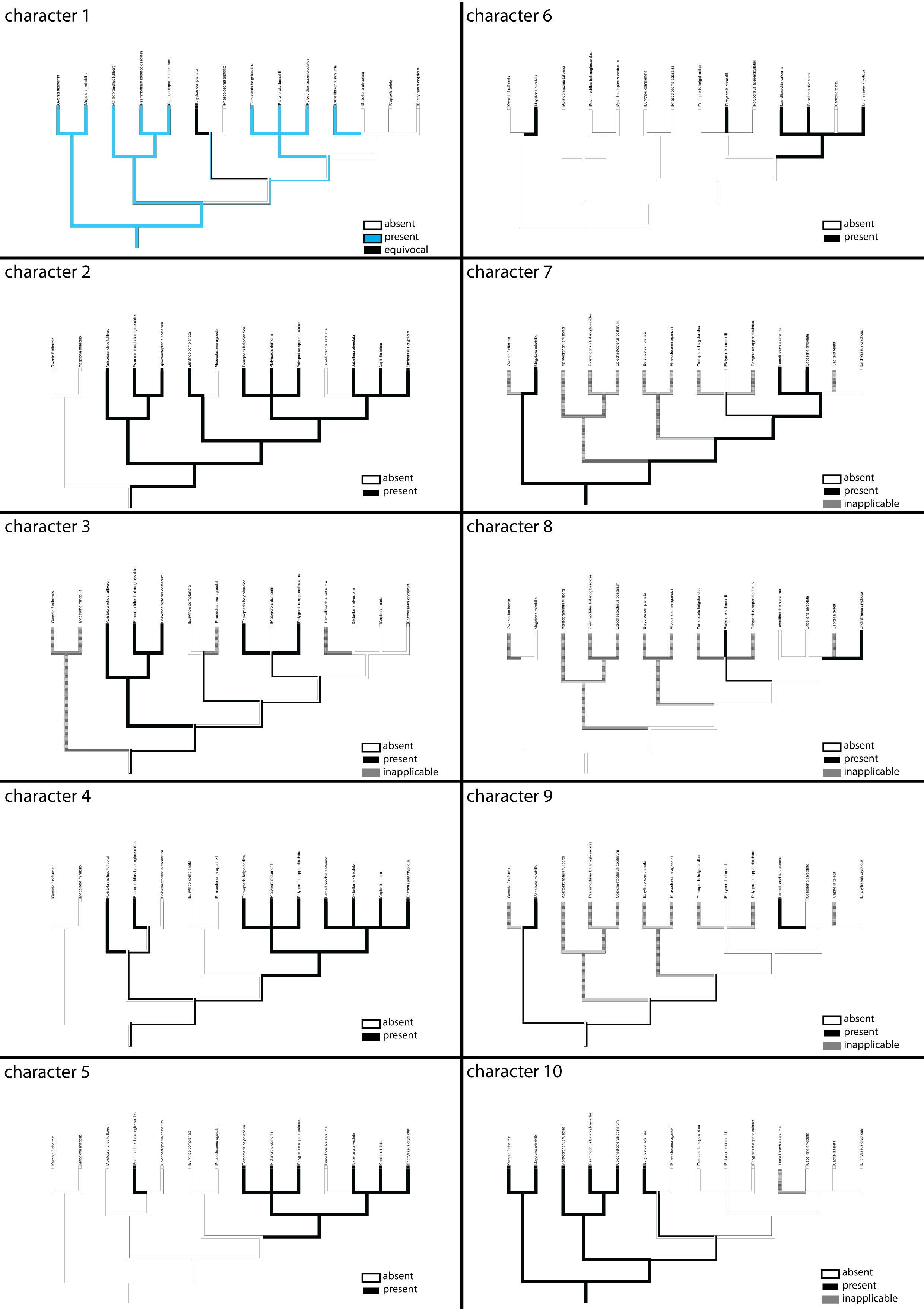

Supplement: Supplementary file 3 — Figure S4. Ancestral state reconstructions for the separate characters of the ventral nerve cord using a parsimony model with characters treated as unordered in MESQUITE v. 3.10. The character state is color coded and shown on the respective branch. (TIF 27174 kb) [file 12983_2018_280_MOESM3_ESM.tif]

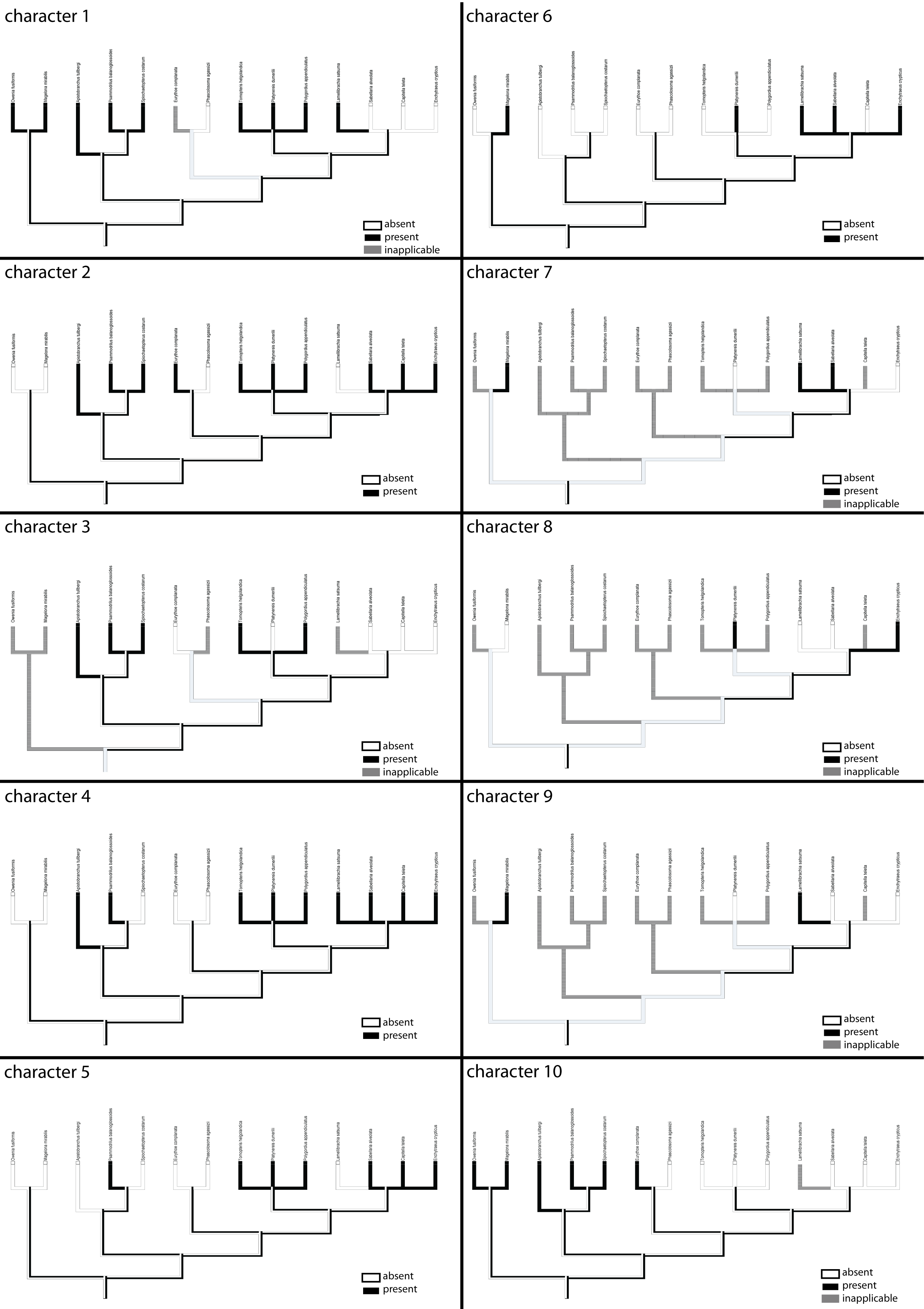

Supplement: Supplementary file 4 — Figure S5. Ancestral state reconstructions for the separate characters of the ventral nerve cord using the maximum likelihood Mk1 model with branch lengths scored as equal in MESQUITE v. 3.10. The character state is color coded and shown on the respective branch. (TIF 27269 kb) [file 12983_2018_280_MOESM4_ESM.tif]

**
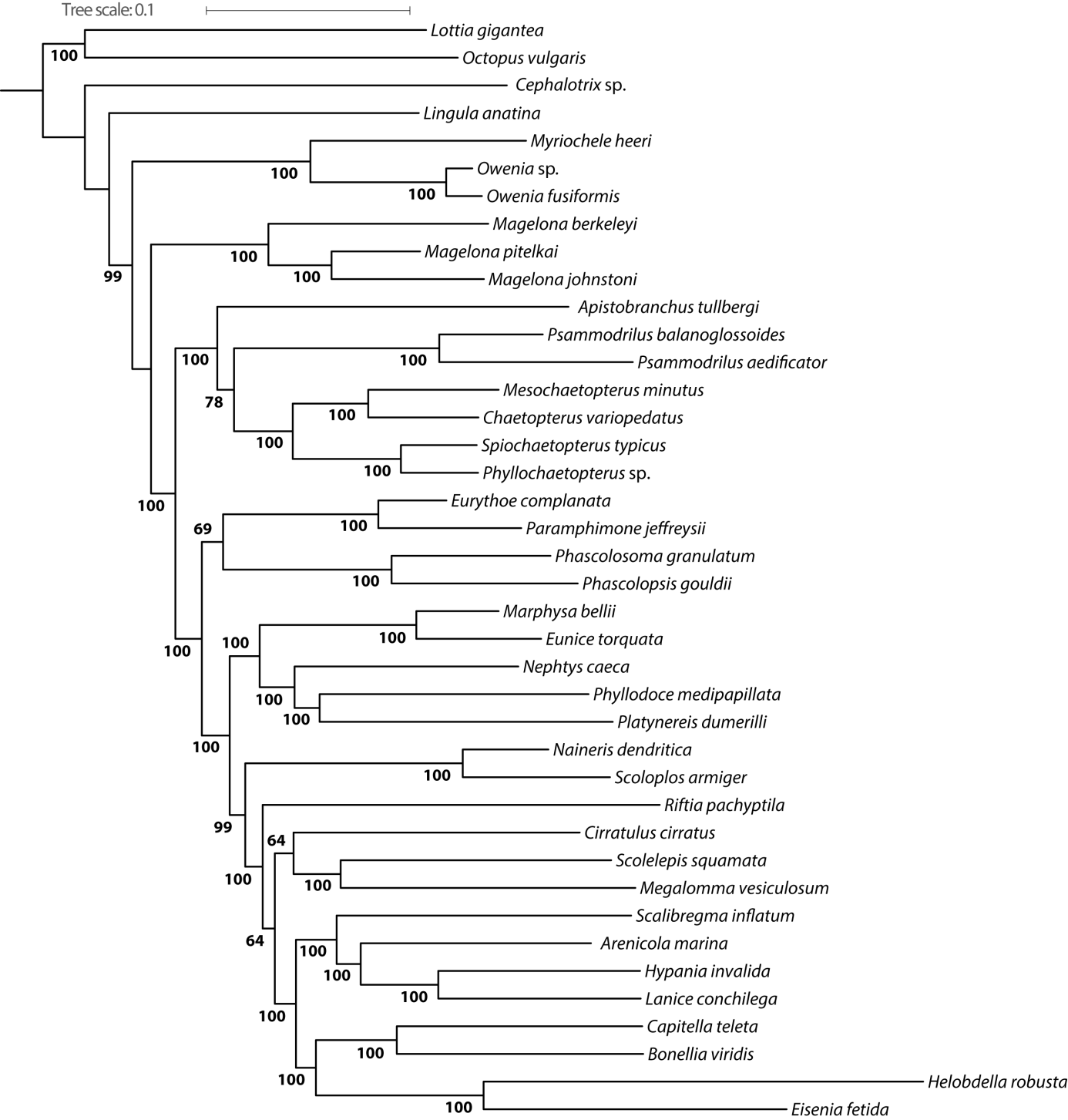
**

Supplement: Supplementary file 5 — Figure S3. Best maximum likelihood (ML) tree of the RAxML analysis using the MARE2 data set of 40 taxa, including 404 gene partitions comprising 128,186 amino acid positions. Only bootstrap values above 50 are shown. (DOCX 250 kb) [file 12983_2018_280_MOESM5_ESM.docx]

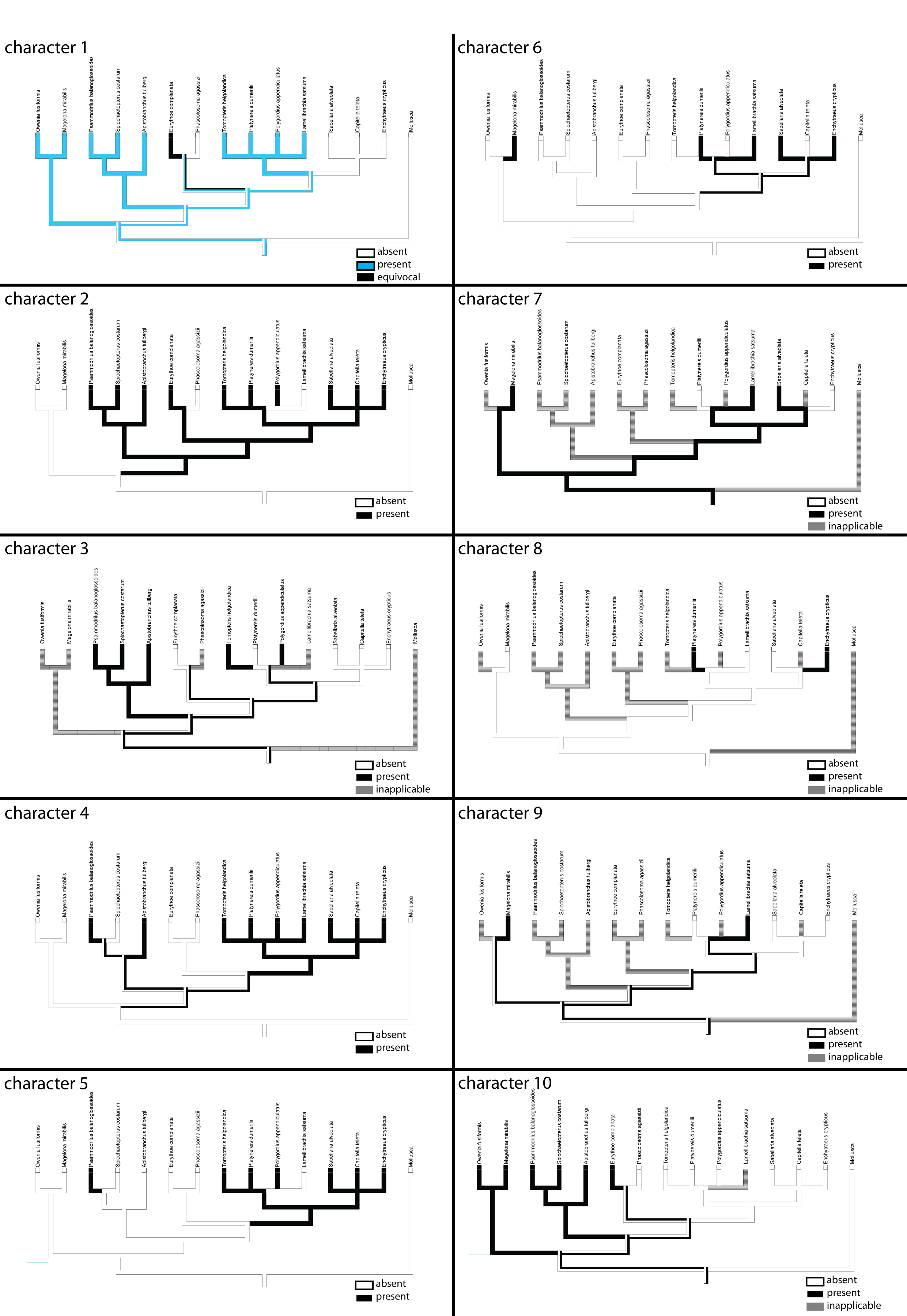

Supplement: Supplementary file 6 — Figure S6. Ancestral state reconstructions for the separate characters of the ventral nerve cord using a parsimony model with characters treated as unordered and Mollusca as outgroup in MESQUITE v. 3.10. The character state is color coded and shown on the respective branch. (TIF 28014 kb) [file 12983_2018_280_MOESM6_ESM.tif]

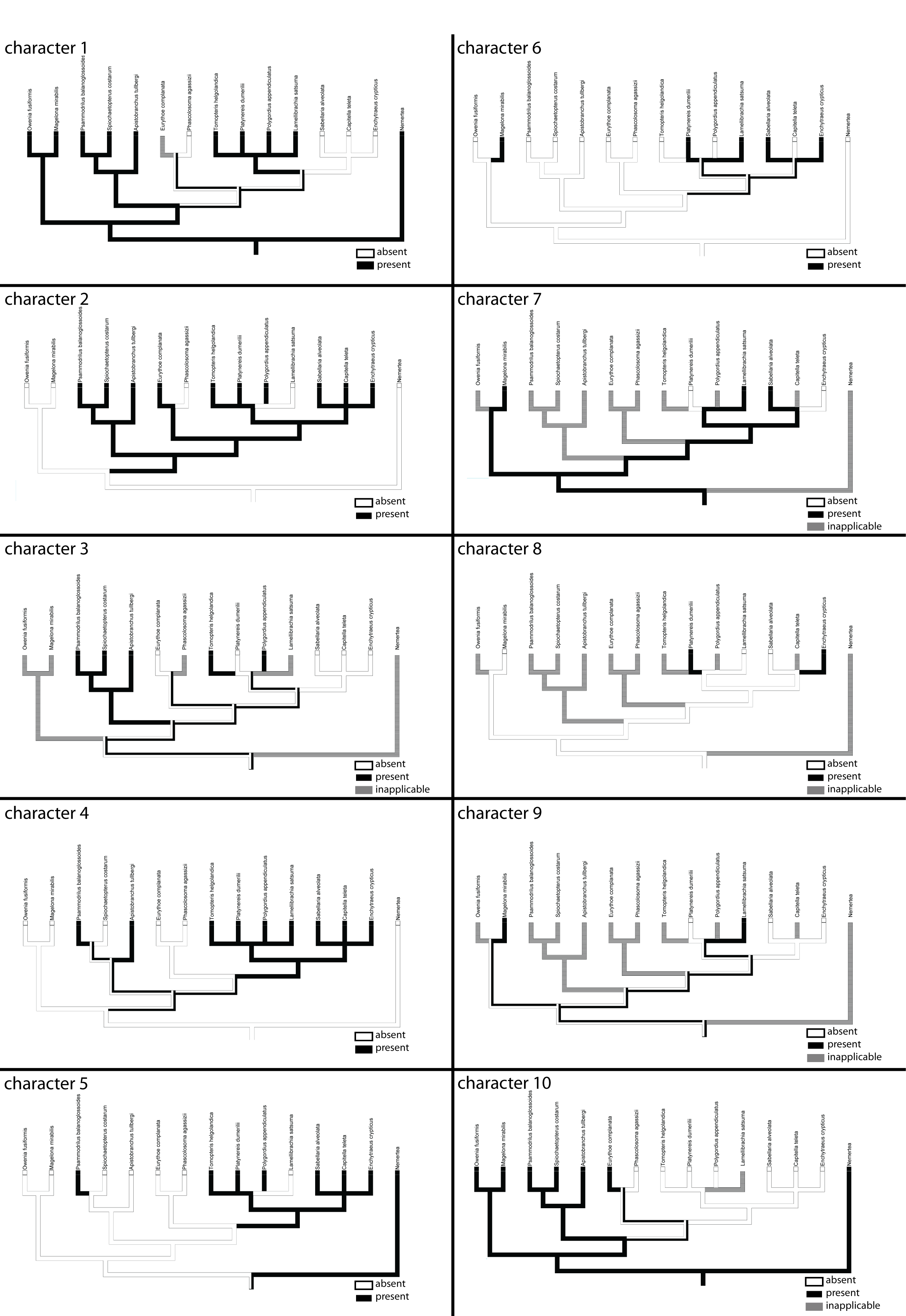

Supplement: Supplementary file 7 — Figure S7. Ancestral state reconstructions for the separate characters of the ventral nerve cord using a parsimony model with characters treated as unordered and Nemertea as outgroup in MESQUITE v. 3.10. The character state is color coded and shown on the respective branch. (TIF 28001 kb) [file 12983_2018_280_MOESM7_ESM.tif]

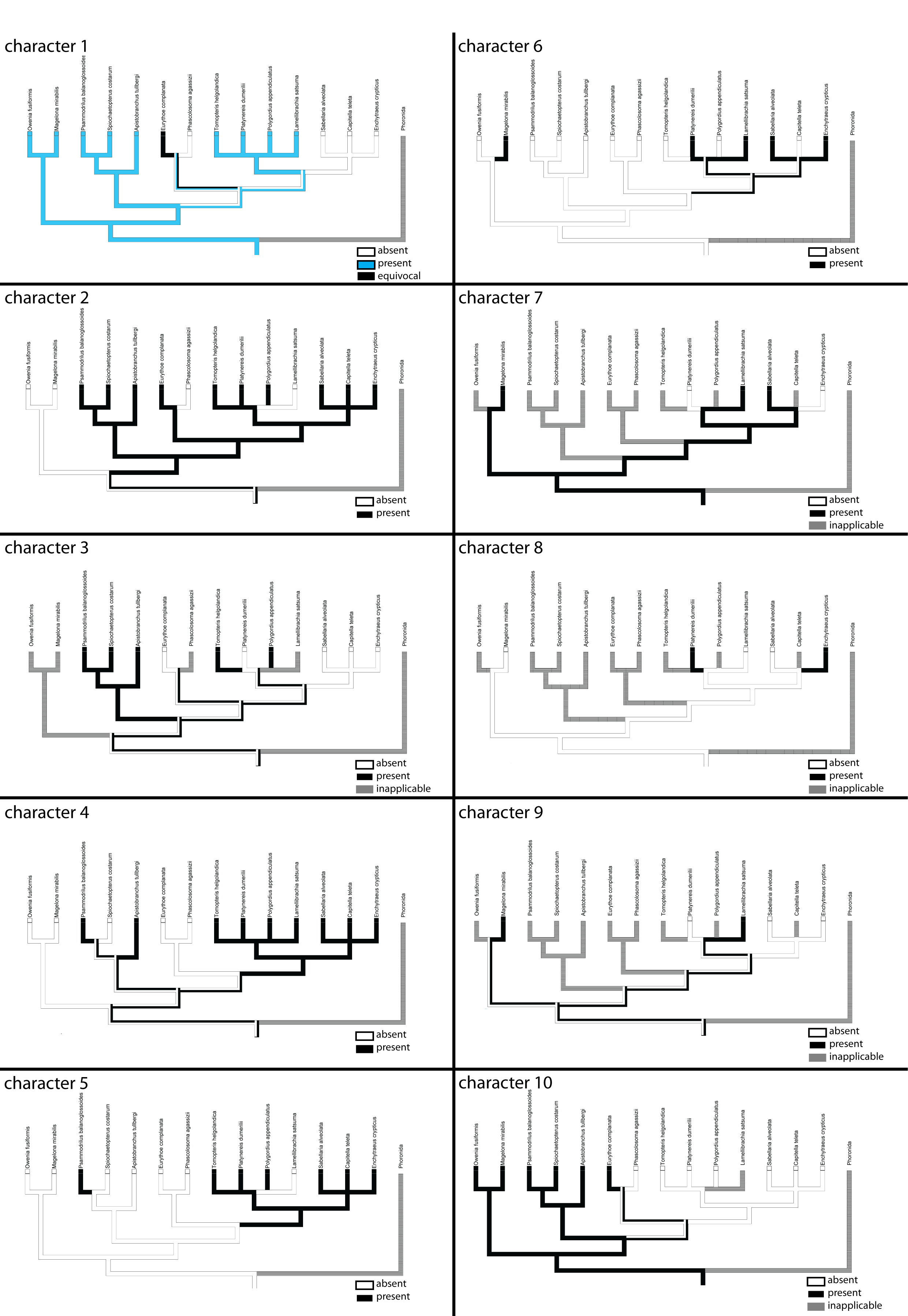

Supplement: Supplementary file 8 — Figure S8. Ancestral state reconstructions for the separate characters of the ventral nerve cord using a parsimony model with characters treated as unordered and Phoronida as outgroup in MESQUITE v. 3.10. The character state is color coded and shown on the respective branch. (TIF 27990 kb) [file 12983_2018_280_MOESM8_ESM.tif]

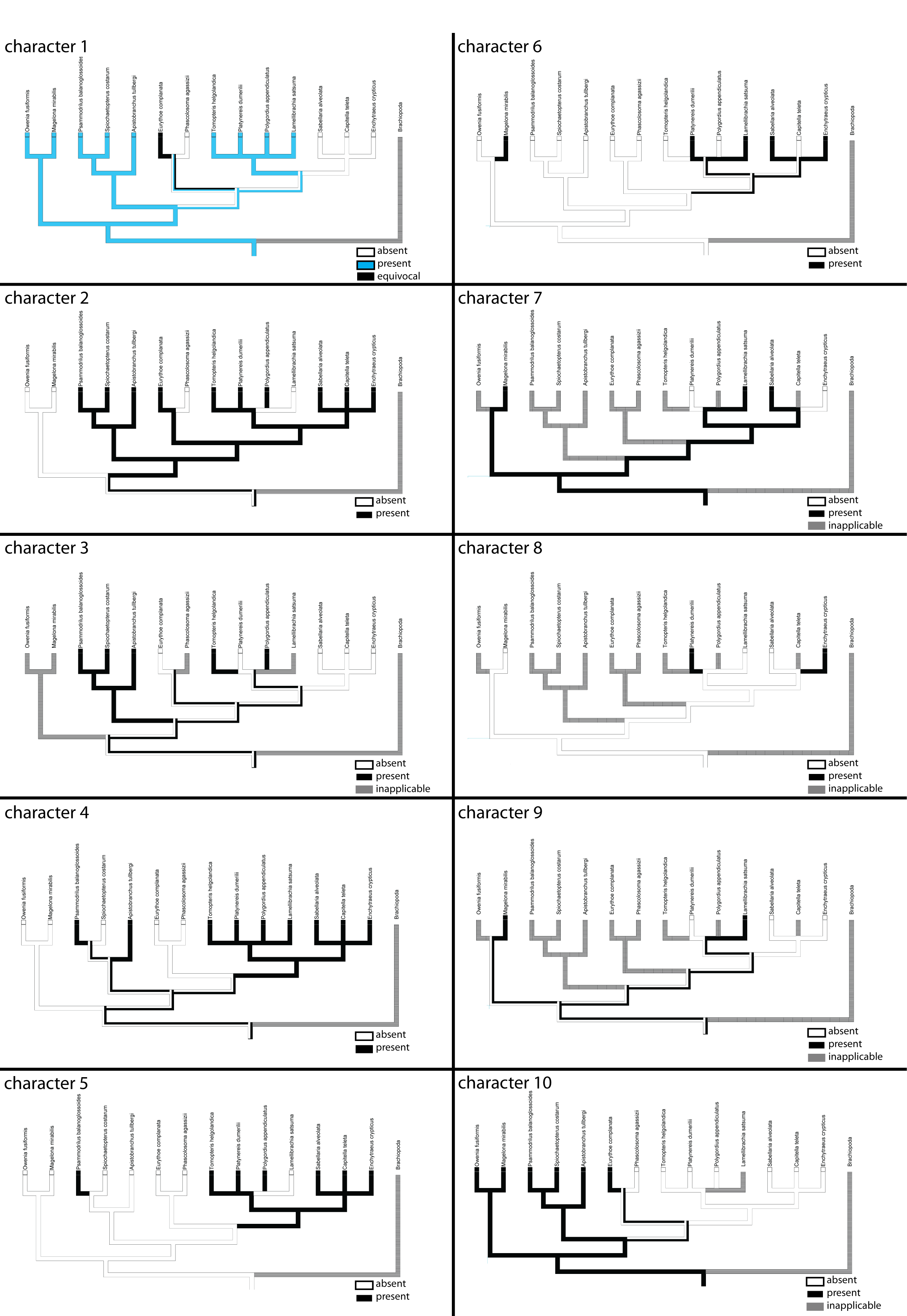

Supplement: Supplementary file 9 — Figure S9. Ancestral state reconstructions for the separate characters of the ventral nerve cord using a parsimony model with characters treated as unordered and Brachiopoda as outgroup in MESQUITE v. 3.10. The character state is color coded and shown on the respective branch. (TIF 28020 kb) [file 12983_2018_280_MOESM9_ESM.tif]
